# The Effect of Alternative Graphical Displays Used to Present the Benefits of Antibiotics for Sore Throat on Decisions about Whether to Seek Treatment: A Randomized Trial

**DOI:** 10.1371/journal.pmed.1000140

**Published:** 2009-08-25

**Authors:** Cheryl L. L. Carling, Doris Tove Kristoffersen, Signe Flottorp, Atle Fretheim, Andrew D. Oxman, Holger J. Schünemann, Elie A. Akl, Jeph Herrin, Thomas D. MacKenzie, Victor M. Montori

**Affiliations:** 1 Norwegian Knowledge Centre for the Health Services, Oslo, Norway; 2 Clinical Research and INFORMAtion Translation Unit, and Department of Epidemiology, Italian National Cancer Institute Regina Elena, Rome, Italy; 3 Department of Medicine, University at Buffalo, Buffalo, New York, United States of America; 4 Division of Cardiology, Yale University, New Haven, Connecticut, United States of America; 5 Department of Internal Medicine, Denver Health and Hospital Authority and University of Colorado Health Sciences Center, Denver, Colorado, United States of America; 6 Knowledge and Encounter Research Unit, Division of Endocrinology and Internal Medicine, Mayo Clinic College of Medicine, Rochester, Minnesota, United States of America; Cardiff University, United Kingdom

## Abstract

**Background:**

We conducted an Internet-based randomized trial comparing four graphical displays of the benefits of antibiotics for people with sore throat who must decide whether to go to the doctor to seek treatment. Our objective was to determine which display resulted in choices most consistent with participants' values.

**Methods and Findings:**

This was the first of a series of televised trials undertaken in cooperation with the Norwegian Broadcasting Company. We recruited adult volunteers in Norway through a nationally televised weekly health program. Participants went to our Web site and rated the relative importance of the consequences of treatment using visual analogue scales (VAS). They viewed the graphical display (or no information) to which they were randomized and were asked to decide whether to go to the doctor for an antibiotic prescription. We compared four presentations: face icons (happy/sad) or a bar graph showing the proportion of people with symptoms on day three with and without treatment, a bar graph of the average duration of symptoms, and a bar graph of proportion with symptoms on both days three and seven. Before completing the study, all participants were shown all the displays and detailed patient information about the treatment of sore throat and were asked to decide again. We calculated a relative importance score (RIS) by subtracting the VAS scores for the undesirable consequences of antibiotics from the VAS score for the benefit of symptom relief. We used logistic regression to determine the association between participants' RIS and their choice. 1,760 participants completed the study. There were statistically significant differences in the likelihood of choosing to go to the doctor in relation to different values (RIS). Of the four presentations, the bar graph of duration of symptoms resulted in decisions that were most consistent with the more fully informed second decision. Most participants also preferred this presentation (38%) and found it easiest to understand (37%). Participants shown the other three presentations were more likely to decide to go to the doctor based on their first decision than everyone based on the second decision. Participants preferred the graph using faces the least (14.4%).

**Conclusions:**

For decisions about going to the doctor to get antibiotics for sore throat, treatment effects presented by a bar graph showing the duration of symptoms helped people make decisions more consistent with their values than treatment effects presented as graphical displays of proportions of people with sore throat following treatment.

**Clinical Trials Registration:**

ISRCTN58507086

*Please see later in the article for the Editors' Summary*

## Abstract 2

In a randomized trial, Cheryl Carling and colleagues evaluate how people respond to different statistical presentations regarding the consequences of taking antibiotic treatment for sore throat.

## Editors' Summary

**Background:**

In the past, patients usually believed that their doctor knew what was best for them and that they had little say in deciding what treatment they would receive. But many modern interventions have complex trade-offs. Patients' opinions about the relative desirability of the possible outcomes of health care interventions depend on their lifestyle and expectations, and these “values” need to be considered when making decisions about medical treatments. Consequently, shared decision-making is increasingly superseding the traditional, paternalistic approach to medical decision-making. In shared decision-making, health care professionals talk to their patients about the risks and benefits of the various treatment options, and patients tell the health care professionals what they expect and/or require from their treatment.

**Why Was This Study Done?:**

Shared decision-making can only succeed if patients know about the treatment options that are available for their medical condition and understand the consequences of each option. But how does the presentation of information about treatment options to patients affect their decisions? In 2002, a series of internet-based randomized trials (studies in which participants are randomly allocated to different “treatment” groups) called the Health Information Project: Presentation Online (HIPPO) was initiated to answer this question. Here, the researchers describe HIPPO 3, a trial that investigates how alternative graphical displays of the benefits of antibiotics for the treatment of sore throat affect whether people decide to seek treatment. In particular, the researchers ask which display results in people making a treatment decision most consistent with their values, i.e., in terms of the relative importance to them of the treatment's desirable and undesirable outcomes.

**What Did the Researchers Do and Find?:**

Adult Norwegians recruited through a television health program numerically rated the importance of symptom relief and of several negative consequences (for example, side effects) of antibiotic treatment for sore throat on the trial's Web site. Relative importance scores (which indicate the participants' values) were calculated for each participant by subtracting their ratings for the importance of the negative consequences of seeking antibiotic treatment from his or her rating for the importance of symptom relief. The participants were then asked to decide whether to visit a doctor for antibiotics without receiving any further information or after being shown one of four graphical displays illustrating the benefits of antibiotic treatment. Two bar charts and one display of happy- and sad-face icons showed the proportion of people with symptoms at specific times after sore throat onset with and without treatment. A third bar chart indicated symptom duration with and without antibiotics. Finally, all the participants were shown all the displays and other information about sore throat and were asked to decide again about seeking treatment. The researchers found a clear association between the participants' values and the likelihood of their deciding to go to the doctor, and this likelihood depended on which graphical display the participants saw. People shown information on the proportion of patients with symptoms were more likely to decide to visit a doctor than those shown information on symptom duration. Furthermore, first decisions reached after being given information on symptom duration or no information were more consistent with the fully informed second decision than first decisions reached after seeing the other displays.

**What Do These Findings Mean?:**

These findings suggest that, for people considering whether to seek antibiotic treatment for sore throat, a bar graph showing the duration of symptoms is more likely to help them make a decision that is consistent with their own values than a bar chart showing the proportions of people with sore throat following treatment. The researchers also found that the bar chart showing symptom duration was preferred by more of the participants than any of the other representations. Whether these results can be applied to other health care decisions or in other settings is not known. However, the researchers suggest that these findings may be most relevant to treatments that, like antibiotic treatment of sore throat, have a short-lived benefit and relatively important downsides.

**Additional Information:**

Please access these Web sites via the online version of this summary at http://dx.doi.org/10.1371/journal.pmed.1000140.

- A *PLoS Medicine* Editorial discusses this trial and the results of another HIPPO trial that are presented in a separate *PLoS Medicine* Research Article by Carling et al.; details of a pilot HIPPO trial are also available
- The Foundation for Informed Medical Decision Making (a US-based nonprofit organization) provides information on many aspects of medical decision making
- The Dartmouth-Hitchcock Medical Center provides information to help people make health care decisions through its Center for Shared Decision Making
- The Ottawa Hospital Research Institute provides information on patient decision aids, including an inventory of decision aids available on the Web (in English and French)
- MedlinePlus provides links to information and advice about sore throat (in English and Spanish)

## Introduction

Relevant, reliable, and accessible information about the effects of interventions is essential for informed choices about health care. The manner in which this information is presented affects how it is understood by both patients and physicians, and their subsequent health care decisions [1]–[3].

The goal of the Health Information Project: Presentation Online (HIPPO) was to improve communication of information about the effects of health care based on randomized trials of alternative ways of presenting evidence of the effects of health care. The current study was the first trial in the series that was presented in cooperation with a weekly health program on the Norwegian Broadcasting Company, with the goal of helping the public to learn about medical research and to use research results to inform their decisions.

The objective of this trial was to determine which format helps people to make decisions that are most consistent with their values. A comparison was made of four graphical displays of the effect of treatment with antibiotics for people with sore throat who must decide whether or not to go to the doctor to get a prescription [4],[5] to find out which format helps people to make decisions that are most consistent with their values. In this context, format includes: “… the visual display aspects as well as … the substantive content dimensions of information” [6]. Values here refers to the relative desirability of the possible consequences of a health care intervention, including health outcomes (such as the discomfort of a sore throat), the burden of treatment (such as the inconvenience of getting and taking antibiotics), and resource expenditures [7].

Previous research has used various constructs to evaluate the effects of graphical displays in communicating treatment effects [6],[8]–[12]. Commonly used graphical displays include bar graphs, pie charts, line graphs, and the use of face icons (i.e., happy/sad). A review of patient comprehension of information found six studies that used graphical displays in a medical decision context [6]. In a comparison of graphical displays among cancer patients, vertical bars, systematic ovals, and numbers resulted in more accurate selection of the larger quantity than horizontal bars, pie charts, and random ovals [8]. Numbers and systematic ovals also resulted in the most accurate estimation of absolute differences between quantities, while random ovals were again worst. In other studies bar charts, thermometer scales and face icons showed no significant quantitative differences with respect to the level of decisional conflict aroused [13],[14] or perceived value of the information to decision making, although bar charts were most commonly preferred [10]. We are not aware of any previous studies that have compared the effects of different graphical displays on the extent to which subsequent decisions were consistent with the decision makers' values.

Thus, we designed this study to assess the extent to which the use of different graphical displays affect choices about whether to go to the doctor for antibiotics for a sore throat. We chose this decision because it is common, familiar to most, and because high-quality evidence informs the benefits and downsides of antibiotics for sore throat [4],[5]. It is a “preference sensitive” decision that is affected by patients' values [15],[16]. Thus, among people with a sore throat, one would expect some degree of correlation between how important the desirable and undesirable consequences of taking antibiotics are to them and the likelihood that they would decide to go to the doctor for antibiotics. In other words, one would expect that people for whom the benefits of taking antibiotics were less important and the downsides more important would be less likely, on average, to decide to go to the doctor than people for whom the benefits were more important and the downsides less important.

## Methods

The CONSORT checklist and the protocol for this study are available as supporting information; see Text S1 and Text S2.

The study was an Internet-based randomized trial in which participants were randomized to one of four graphical displays of information about the effects of antibiotics on the symptoms of sore throat or to no information (see flow diagram in Figure S1). The objective was to compare the impact of the graphical displays on decisions about whether to go to the doctor for antibiotics in relation to the values of the participants. We used estimates of the effects of antibiotics for sore throat for *Streptococcus*-positive, *Streptococcus*-negative, and untested patients from a systematic review [4]

### Interventions and Comparisons

**Figure 1:**
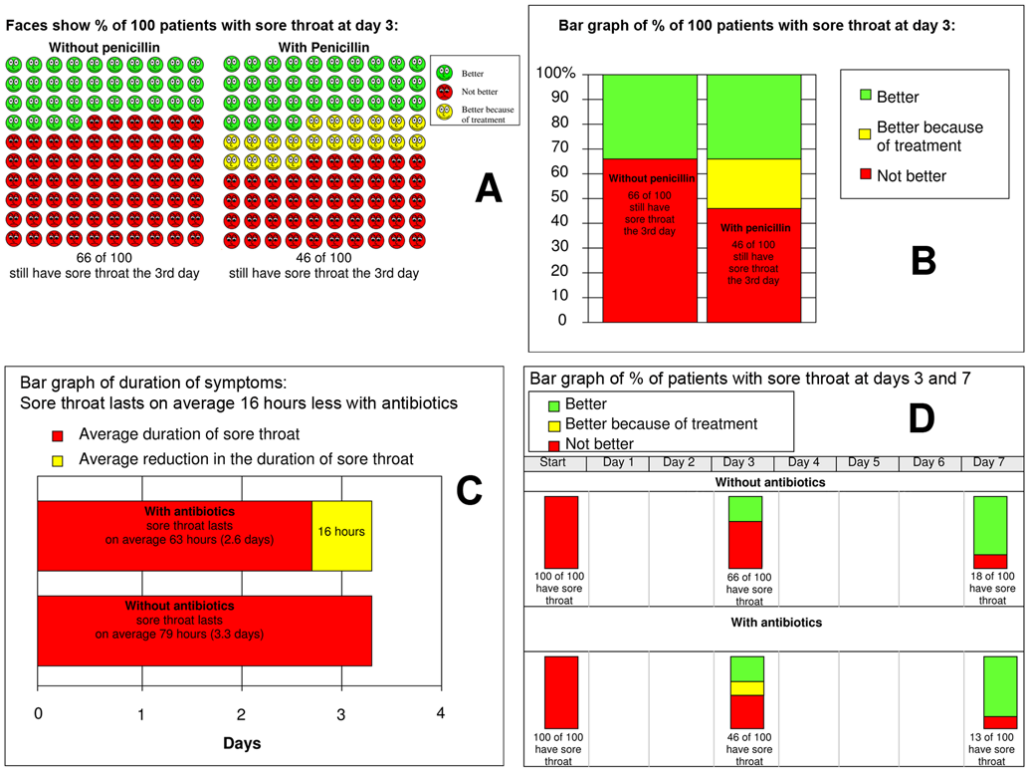
Presentations of benefits of antibiotics for sore throat. Based on a systematic review by Del Mar and colleagues [4] of antibiotic versus placebo for patients presenting for primary care with symptoms of sore throat.

We evaluated these four graphical displays: (1) face icons using happy and sad expressions displaying the proportion of people who still have sore throat symptoms on day three, (2) a bar graph displaying the same information, (3) a bar graph displaying the difference in the average duration of symptoms, and (4) a bar graph displaying the proportion of people who have sore throat symptoms at onset, on day three, and on day seven (Figure 1).

Bar graphs are widely used and familiar to most people. The use of icons, such as faces, has become popular more recently, particularly in the context of decision support tools. We initially considered using a line graph (survival curves for the duration of symptoms). This display contains the most information relative to the other alternatives, but following consultations with colleagues, we concluded that this would be difficult for many people to understand. We therefore elected to use a second bar graph presentation that includes similar information to that presented in a line graph. Day three is the point of maximum benefit and by day seven most people no longer have symptoms with or without antibiotics.

We planned three main comparisons in advance: (1) different displays with the same information—face icons versus the bar graph, both displaying the proportion of people who have sore throat symptoms on day three, (2) the same display with different information—the bar graph displaying the difference in the average duration of symptoms versus the bar graph displaying the proportion of people who have sore throat symptoms on day three, and (3) the same display with additional information—the bar graph displaying the proportion of people who have sore throat symptoms on both day three and day seven versus the bar graph displaying day three only.

### Study Design

Information about the study was broadcast on Puls, a popular nationally televised weekly health program with approximately 700,000 viewers (total population of Norway = 4.5 million). We presented documentation of wide variation in the use of antibiotics for sore throat in Norway on the program and then invited viewers to go to our Web site to participate in the study. The Web site was in Norwegian.

**Figure 2:**
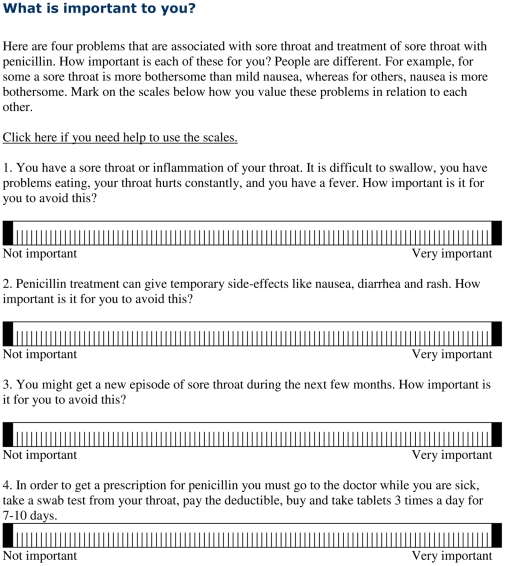
Value elicitation.

Upon logging into the Web site, participants were presented with information about the study and asked to give informed consent. They viewed a brief scenario in which they were asked to imagine that they had a sore throat and needed to decide if they would go to the doctor for antibiotics. Participants were then requested to indicate the relative importance of the discomfort of a sore throat, side effects of antibiotics, recurrence of sore throat, and the inconvenience of getting and taking antibiotics using horizontal 100-point visual analogue scales (VAS) (Figure 2). The lower and upper anchors of the VAS were labeled “Not important” and “Very important.”

**Figure 3:**
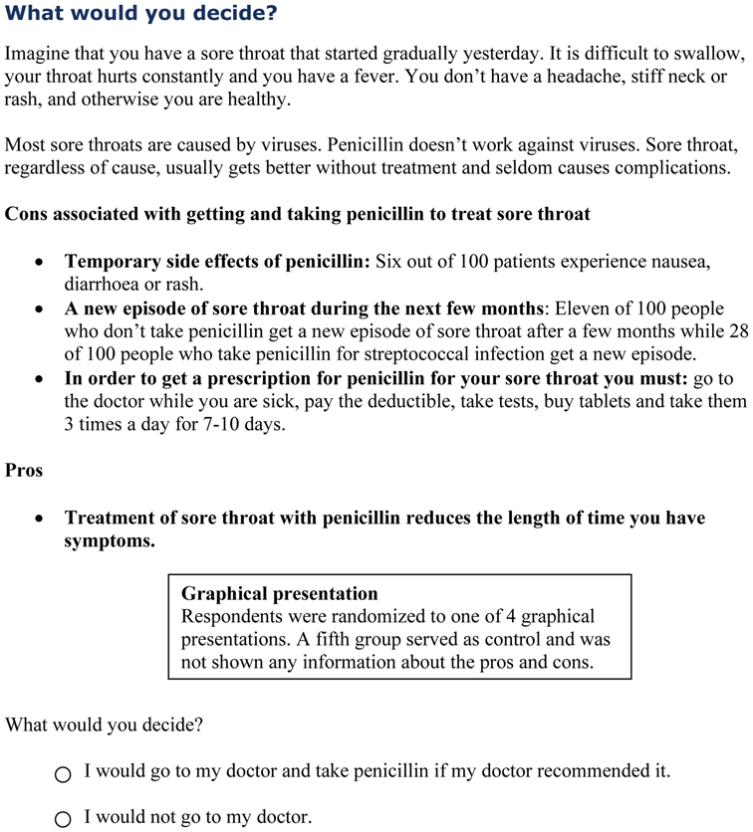
Downsides of antibiotics and decision elicitation.

They then viewed one of the four graphical presentations or received no information, based on random allocation. The system randomized participants upon log-on, using block randomization with a looped sequence of 500 presentation assignments consisting of 100 blocks of five that was generated on http://www.randomization.com. Participants who had been randomized to one of the graphical displays were all shown the same textual information about the downsides of taking antibiotics for sore throat while the benefit of taking antibiotics was presented by the allocated graphical display (Figure 3). Next, participants were asked to indicate whether they would or would not go to the doctor for antibiotics (Figure 3). They were then asked a few questions about themselves. Afterward, all participants were shown all the presentations in a block-randomized sequence, and were asked which presentation they preferred and which was easiest to understand. They were then shown detailed evidence-based patient information about the causes and treatment of sore throat from a previous study [17] and asked to reconsider their original decision and decide again if they would go to the doctor. Our premise was that the more fully informed second decision could serve as a benchmark with which the original decisions could be compared.

Responses from participants who stated they were at least 18 years old and that they were filling in the questionnaire for the first time were included in the analysis. Participants' responses to the questions on our Web site were saved directly into a database where the data were stored anonymously. Confidentiality of data was ensured by not collecting any information that would make it possible to identify the participants. Voluntary contact information that some participants supplied in order to be informed of future studies was stored in a separate database so it was not possible to couple contact information and study data. Participants were informed on the consent screen that they could leave the study at any time and were given the option of choosing to have any data that they might have entered deleted.

### Analysis and Sample Size

For each participant, we calculated a relative importance score (RIS) by subtracting the sum of her VAS scores for the relative importance of avoiding the downsides of antibiotics (side effects; recurrence of sore throat, which is greater with antibiotic treatment; and the inconvenience of getting and taking antibiotics) from her VAS score for the relative importance of avoiding the discomfort of a sore throat. We expected that higher RIS would be correlated with an increased likelihood of deciding to go to the doctor.

In order to compare the effects of the different graphical displays on the decision to go to the doctor, taking into account each participant's RIS, we used logistic regression with the decision to go to the doctor (yes or no) as the dependent variable, and the RIS and allocated display as predictors. The following model was used:
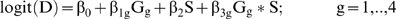
where D is the decision to go to the doctor or not, G is the presentation group, S is the RIS value, and G*S is the interaction between the presentation and the RIS value. To make inferences about the response within each group and for the comparisons of groups, we used dummy variable coding with reference parameterization for the presentation groups, i.e., directly estimating the difference in the effect between the presentation groups and the reference group, i.e. bar graph at day 3. Wald tests were used for the *p*-values and confidence intervals from the logistic regression, whereas Chi-square tests were used for comparison of frequencies.

Based on the results of previous studies [18],[19], we estimated we would need about 600 participants per group to achieve 80% power for each of the three main comparisons (comparing the linear predictors of the groups) at alpha level 0.0167 after applying a Bonferroni correction.

Additional comparisons for the difference in log odds at the 1st and 3rd quartiles and the median values of RIS were planned.

We considered which group made decisions that were the most consistent with the “more fully informed” second decision, after participants have seen all four presentations and been provided more detailed information. This was done by comparing the linear predictors for each group for the first decision with the linear predictor (pooled estimate) for the other four groups for the second decision using the model above without the interaction term, which was not statistically significant. We also counted the changes from the first to the second decision in each group. We used a logistic regression model to explore whether the respondents did or did not change their decisions depending on the RIS, presentation group, and the interaction.

## Results

The trial was conducted September–October 2004 and stopped when recruitment tapered off after three weeks. There were 1,760 participants after excluding those under 18 (see flow diagram, Figure S1). The five groups were similar with respect to sex, age, education, and VAS scores for their values (Table 1). Sixty-nine percent were women, compared to 51% in the Norwegian population. A larger proportion of participants were under 40, a smaller proportion over 50, and a larger proportion had university level education, compared to the general population.

**Table 1:** Participant characteristics.

Category	Subcategory	Face icons, % at Day 3	Bar Graph, % at Day 3	Bar Graph, Duration of Symptoms	Bar Graph, % at Days 3 and 7	No Information	Total	Norwegian Population^a^
		*n* = 361	*n* = 355	*n* = 351	*n* = 319	*n* = 374	*N* = 1,760	
**Women** b		70.9	70.7	67.2	67.1	70.9	69.4	51.0
**Age** b	18–29	25.8	28.7	26.8	28.8	31.6	28.4	19.4
	30–39	32.7	33.0	32.2	30.1	30.5	31.7	20.0
	40–49	22.4	21.7	22.2	20.4	19.8	21.3	18.3
	50–59	15.0	13.2	12.0	15.4	13.6	13.8	17.0
	60–69	3.9	2.8	6.0	4.4	2.9	4.0	10.7
	70–79	0.3	0.6	0.6	0.9	1.3	0.7	8.6
	Over 80	0	0	0.3	0	0.3	0.1	6.0
**Education** b	Elementary	3.0	4.5	2.3	3.8	3.7	3.5	31.0
	High school	22.4	26.2	24.8	21.0	25.1	24.0	42.7
	University	74.5	69.3	72.9	75.2	71.1	72.6	23.3
**Values (on 100-point visual analogue scale)** c	Sore throat	69.4 (26.0)	74.1 (24.3)	72.1 (25.1)	69.2 (25.1)	70.5 (25.8)	71.1 (25.3)	
	Side effects	63.9 (29.7)	63.1 (31.4)	62.1 (31.3)	65.3 (28.8)	64.4 (31.1)	63.7 (30.5)	
	Recurrence	76.8 (24.0)	79.6 (21.5)	77.1 (25.6)	77.2 (24.1)	75.5 (26.1)	77.2 (24.4)	
	Inconvenience	64.1 (32.6)	63.2 (33.3)	61.4 (33.3)	63.8 (31.9)	62.0 (34.5)	62.9 (33.2)	
	RIS	−135.4 (55.4)	−131.9 (60.6)	−128.5 (63.7)	−137.2 (59.2)	−131.4 (59.1)	−132.8 (59.7)	

a For the Norwegian population the proportion of women and each age group is based on the population over 17 in 2004 [25]. The proportion of people with different levels of education is based on the highest completed education for people over 16 years old [26].

b Data presented as percentages of *n* in a given column.

c Data presented as mean (standard deviation) for a given presentation group.

Overall, 27.7% of participants chose to go to the doctor on the first decision (Table 2). There were statistically significant differences across the five groups (*p*<0.0001). The group that viewed the bar graph of duration of symptoms had the smallest proportion that would go to the doctor (19.7%), closely followed by the group that received no information (22.7%). The groups that viewed the faces at day 3 and the bar graph at day 3 had the largest proportion of people who would go to the doctor (34.6% and 34.4 % respectively).

**Table 2:** Decisions to go to the doctor.

Decision	Face Icons, % at Day 3	Bar Graph, % at Day 3	Bar Graph Duration of Symptoms	Bar Graph, % at Days 3 and 7	No Information	Total	*p*-Value
**First decision**							
Responses	(361)	(355)	(351)	(319)	(374)	(1,760)	
Would go to doctor	34.6 (125)	34.4 (122)	19.7 (69)	27.3 (87)	22.7 (85)	27.7 (488)	<0.0001
**Second decision**							
Responses	(344)	(337)	(339)	(310)	(355)	(1,685)	
Would go to doctor	24.1 (83)	27.0 (91)	18.6 (63)	24.5 (76)	17.5 (62)	22.3 (375)	0.01
**Change from first to second decision**							
From “go” to “not go”	34.5 (41/119)	27.6 (32/116)	23.4 (15/64)	18.8 (16/85)	36.7 (29/79)	28.7 (113/463)	0.052
From “not go” to “go”	2.2 (5/225)	3.2 (7/221)	5.1 (14/275)	3.1 (7/225)	4.3 (12/276)	3.7 (45/1,222)	0.462
Total changes	13.4 (46)	11.6 (39)	8.6 (29)	7.4 (23)	11.5 (41)	10.6 (178)	0.81

Data are presented as percentages of number in group.

Overall, 22.3% decided to go to the doctor on the second, more fully informed decision (Table 2). Among those who first answered positively, 28.7% changed their decision from going to not going, compared to only 3.7% that changed in the opposite direction. The proportion of participants who changed their decision from going to not going ranged from 36.7% in the group first shown no information to 18.8% in the group first shown the bar graph at days 3 and 7 (p = 0.052).

The largest number of participants (38.4%) preferred the bar graph of duration, followed by the bar graph at day 3 (30.4%), while the fewest preferred the faces at day 3 and the bar graph at days 3 and 7 (14.4% and 16.8%, respectively) (*p*<0.0001). Similarly, most participants (37.4%) found the bar graph of duration easiest to understand, followed by bar graph at day 3 (29.7%), while the fewest found the faces at day 3 and the bar graph at days 3 and 7 easiest to understand (14.0% and 16.4%, respectively).

### Decisions in Relation to Values

**Figure 4:**
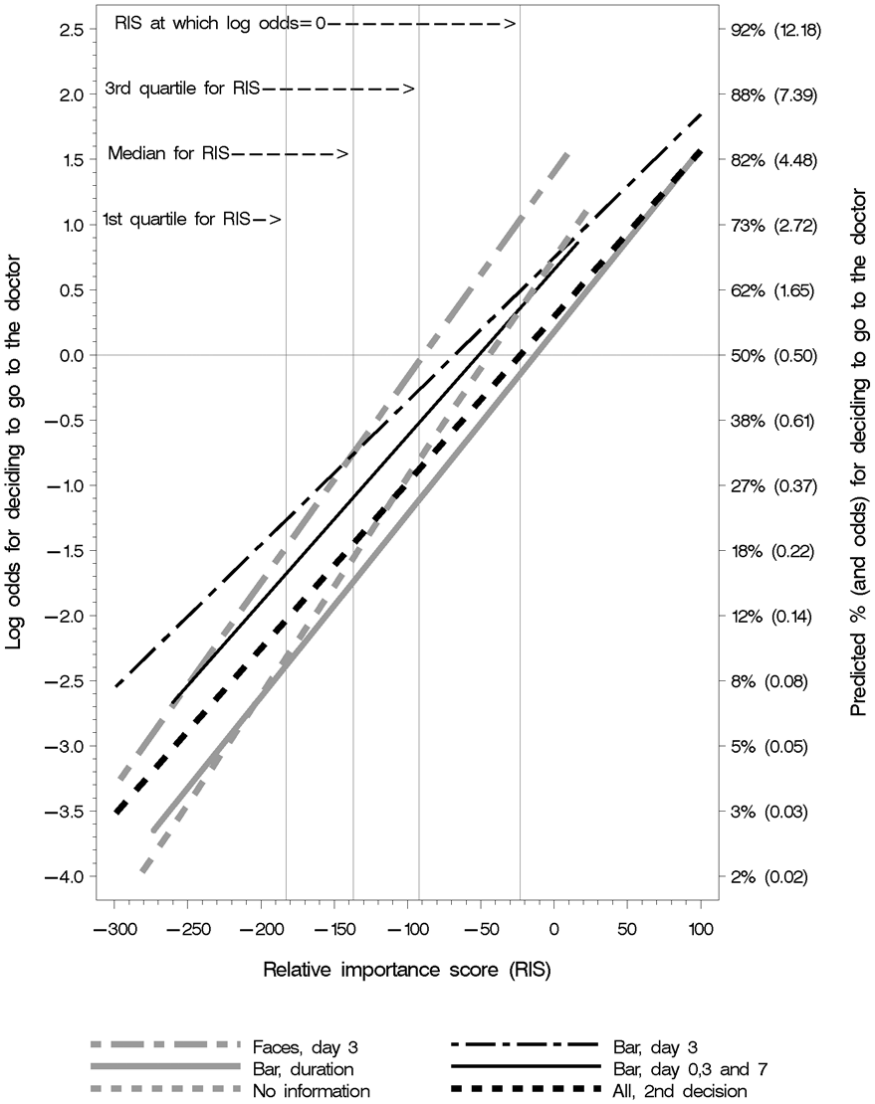
Likelihood of deciding to go to the doctor in relation to RIS. Relative importance score (RIS) values indicate the relative importance to participants of the desirable and undesirable consequences of getting and taking antibiotics. As anticipated, the likelihood of participants deciding to go to the doctor is greater when the relative importance of the desirable consequences (a shorter duration of sore throat) is greater and the relative importance of the downsides of getting and taking antibiotics is less.

There was a clear association between the participants' elicited values (estimated using the RIS) and the likelihood of their deciding to go to the doctor (Figure 4). As the RIS increased, the probability of deciding to go to the doctor increased. The groups shown the faces or the bar graph at day-3 were most likely to decide to go to the doctor at the median and the 1^st^ and 3^rd^ quartiles of the RIS values (Table 3). The group shown the bar graph at days 3 and 7 was slightly less likely to decide to go to the doctor, whereas the group shown the bar graph of duration of symptoms was consistently least likely to decide to go to the doctor. The likelihood of deciding to go to the doctor for the group given no information was similar to the group shown the bar graph of duration of symptoms. The interaction between the RIS and presentation group was not statistically significant (*p* = 0.46) in the logistic regression model (Table 4). Thus the null hypothesis of equal slope of the linear predictors was not rejected, and we therefore report the odds ratios (ORs). The largest difference between the groups was for the bar graph of duration compared to the bar graph at day 3, equivalent to OR = 0.39 (95% CI 0.27 to 0.57) (Table 4). An increase of 10 units in RIS increased the odds by 14.9%.

**Table 3:** Likelihoods for deciding to go to doctor in relation to values (RIS).

Presentation	1^st^ Quartile		Median		3rd Quartile	
	RIS = −183		RIS = −137		RIS = −92	
	Odds (95% CI)	Predicted % (95% CI)	Odds (95% CI)	Predicted % (95% CI)	Odds (95% CI)	Predicted % (95% CI)
**Face icons, % at day 3**	0.23 (0.16–0.33)	18.6 (13.8–24.5)	0.47 (0.37–0.60)	32.0 (27.0–37.4)	0.95 (0.72–1.26)	48.8 (41.9–55.8)
**Bar graph, % at day 3**	0.28 (0.20–0.39)	21.9 (16.7–28.2)	0.47 (0.37–0.59)	31.8 (26.9–37.1)	0.76 (0.59–0.99)	43.4 (37.1–49.8)
**Bar graph, duration of symptoms**	0.09 (0.06–0.15)	8.4 (5.4–12.9)	0.18 (0.13–0.24)	14.9 (11.2–19.6)	0.33 (0.25–0.44)	24.7 (19.8–30.4)
**Bar graph, % at days 3 and 7**	0.19 (0.13–0.27)	15.7 (11.2–21.5)	0.33 (0.26–0.44)	25.1 (20.3–30.5)	0.59 (0.44–0.79)	37.3 (30.7–44.3)
**No information**	0.10 (0.06–0.15)	8.9 (5.8–13.4)	0.21 (0.16–0.29)	17.4 13.4–22.2)	0.45 (0.34–0.59)	30.9 (25.5–37.0)
**Second decision (all)**	0.13 (0.11–0.16)	11.5 (9.7–13.7)	0.23 (0.21–0.27)	19.0 (17.0–21.1)	0.42 (0.37–0.47)	29.4 (26.8–32.1)

Predicted % = proportion deciding to go to the doctor based on logistic regression.

**Table 4:** Comparisons of the presentation groups.

Presentation	Odds Ratio (98.3% CI)^a^	*p*-Value
**Face icons % at day 3 versus bar graph % at day 3**	1.08 (0.78–1.50)	0.65
**Bar graph duration of symptoms versus bar graph % at day 3**	0.39 (0.27–0.57)	<0.001
**Bar graph % at days 3 and 7 versus bar graph % at day 3**	0.74 (0.52–1.05)	0.10

a Adjusted overall CI level = 0.95.

Because the interaction term was not statistically significant and the differences in slopes (Figure 4) may be due to chance, we removed the interaction term to compare the decisions made by each group to the more fully informed second decision made by everyone (Table 5). This model assumes that the slopes are the same. We then compared the odds of deciding to go to the doctor based on the first decision for each group with the odds of deciding to go to the doctor based on the second decision for the pooled results for the other four groups (Table 5). The only groups that were not more likely to decide to go to the doctor on the first decision were the group that was first presented the bar graph of duration of symptoms (OR 0.72; 95% CI 0.53 to 0.98) and the group that was not shown any information for the first decision (OR 0.93; 95% CI 0.70 to 1.24). The ORs for the other three groups were all statistically significant (Table 5).

**Table 5:** Odds ratios for deciding to go to the doctor on the first decision for each group compared to the more fully informed second decision for the other four groups.

Presentation for the First Decision	Odds Ratio (95% CI)
Face icons, % at day 3	2.20 (1.68–2.88)
Bar graph, % at day 3	2.08 (1.59–2.73)
Bar graph, duration of symptoms	0.72 (0.53–0.98)
Bar graph, % at days 3 and 7	1.50 (1.11–2.01)
No information	0.93 (0.70–1.24)

There were no statistically significant differences across the different presentation groups in the proportions of participants who changed their initial decision (Table 2). The logistic regression of whether the respondents changed their decision or not, depending on the RIS, presentation group, and the interaction, indicated a marginal interaction between the RIS and the presentation group shown the faces (*p* = 0.04). Thus, a simple logistic regression of the change, depending on the RIS, was performed per group. The change for the group shown faces was significant (*p* = 0.001, for testing β_2_ = 0) and for the group given no information (*p* = 0.02, for testing β_2_ = 0). This suggests that the respondents changed their mind irrespectively of their RIS when shown the additional information.

## Discussion

In the course of 26 days (13 September to 8 October 2004), there were 4,053 log-ons to the study Web site, resulting in 1,760 usable records. TV recruitment was substantially more successful than the methods we used in previous studies, including the use of commercial email lists and advertising [18],[19]. In those studies it took two years to recruit just under 3,000 participants using other methods [19].

The randomization process generated five comparable groups. It is not possible to say how similar or different participants' decisions were relative to the general population of Norway. Participants were more likely to be female and younger and to have a higher education than the general population.

The participants largely decided not to go to the doctor for antibiotics, regardless of the information they received. Overall, 78% said they would not go to the doctor after seeing all four presentations and receiving more detailed information about the advantages and disadvantages associated with getting and taking antibiotics for sore throat.

### Different Displays with the Same Information

We compared two different visual displays in this study: bar graphs and faces, both showing the proportions of people with symptoms at day 3 with and without antibiotics. The proportions of participants deciding to go to the doctor in relationship to their values (RIS) were similar for the two groups shown these two displays (Table 3), with no significant differences (Table 4).

However, the fewest participants preferred face icons at day 3 or found that display easiest to understand (14%). The group shown the display of face icons at day 3 were also most likely to change (34%) from a positive to a negative decision about going to the doctor after receiving additional information, including all four displays. These findings are consistent with those of Edwards and colleagues in a randomized trial of Web-based information for people with diabetes [9]. They found that bar charts were most commonly preferred and least often found difficult, whereas face icons were more likely to be found unhelpful or patronizing.

### The Same Display with Different Information

Our second comparison was of the same display (bar graphs) with different information: the proportions of people with symptoms at day 3 and the average duration of symptoms with and without antibiotics. Of the three comparisons in this study, this is the only one where we found statistically significant differences when comparing odds at specific RIS values. Participants in all of the groups were more likely to decide to go to the doctor as their RIS increased (Figure 4), as would be expected. However, participants shown the duration of symptoms were less likely to decide to go to the doctor across RIS values (OR 0.33 to 0.43), with predicted differences of 13%–19% (Table 3). These differences correspond to number needed to treat of 5–8; i.e. for every 5–8 participants shown the proportions of people with symptoms at day 3, one additional participant chose to go to the doctor, compared to those shown the duration of symptoms.

There are at least two possible explanations for this difference. One is that the effectiveness of antibiotics appears smaller when considering the average difference in duration of symptoms (63 h versus 79 h) than when considering the difference in the proportion of people with symptoms at day 3 (46% versus 66%) (Figure 1). A second possible explanation is that participants found this display easier to understand. We also cannot rule out that small differences in the displays played a role: vertical versus horizontal bar graphs and three categories in the “with antibiotics” bar (better, better because of treatment, and not better) versus two categories (duration of symptoms and the difference in duration compared to not taking antibiotics).

Of the four presentations, the bar graph of duration of symptoms resulted in decisions that were most consistent with the more fully informed second decision (Table 5). Most participants also preferred this presentation (38%) and found it easiest to understand (37%). Participants shown the other three presentations were more likely to decide to go to the doctor based on their first decision than everyone based on the second decision.

### The Same Display with Additional Information

Our third comparison was of the same display with additional information: bar graphs displaying the proportion of people with sore throat at both day 3 and day 7 versus only at day 3. The proportions of participants deciding to go to the doctor in relationship to their values (RIS) were 6.1% to 6.7% less for the group shown bar graphs for both days 3 and 7 (Table 3), but the ORs for this comparison (0.66 to 0.78) were not statistically significant (Table 4).

Because most people do not have symptoms by day 7 we anticipated that fewer participants would decide to go to the doctor when shown this additional information. A potential explanation for why the observed differences were small and inconclusive is that participants found the display with the additional information difficult to understand.

The group shown the bar graph at days 3 and 7 was less likely to change from a positive to a negative decision (Table 2). We also anticipated this, given the additional information provided in this display, showing that most people are better with or without antibiotics by day 7. However, this display was the second least preferred (17%), and the second fewest participants found this display easiest to understand (16%). The low preference rating for this display might have been due to information overload, although in a previous study participants preferred a presentation with multiple time points for long-term scenarios [20].

### No Information versus Some Information

In this study we used a second more fully informed decision as a benchmark with which the original decisions could be compared. The group that was not shown any information for their first decision was the most likely to change their decision from going to not going to the doctor, although these differences were not statistically significant (*p* = 0.052) (Table 2). The proportion of participants in the “no information” group that changed their decision from “to go” to “not to go” to the doctor suggests that a number of participants (8%) likely started out assuming that the desirable consequences outweighed the undesirable consequences and changed their minds when shown the information in Figures 1 and 3.

Nonetheless, decisions taken by participants in the “no information” group appear to be closest to those taken by the group shown the duration of symptoms and by all of the participants for the second “more fully informed” decision (Table 3). This suggests that the information that was presented confirmed what most people assumed about the trade-offs between the desirable and undesirable consequences of getting and taking antibiotics, and that the presentations showing the proportions of people with and without symptoms may have to some extent “misinformed” participants relative to their second “more fully informed” decision.

### Applicability of the Findings and Implications

The participants were recruited through a popular nationally televised weekly health program and needed to have access to the Internet. Compared to the general population they had more education (Table 1). It is unclear whether the findings are applicable to populations with less education [2],[18] or to other countries, although the results are likely widely applicable in Norway. It is also uncertain to what extent results from the hypothetical scenario used in this study apply to actual decisions [21],[22], although it is likely that most of the participants would have experienced sore throat and thus be able to make a realistic assessment of what they would actually decide. The results are more directly applicable to patient information accessed over the Internet, but it seems likely that the differences in decisions between the groups presented information about the proportion of people with symptoms at day 3 and the duration of symptoms is relevant to personal communication as well as to electronic and printed information.

Large variation exists in the extent to which antibiotics are prescribed for sore throat [17]. Clinical practice guidelines for the management of sore throat also vary with regard to the choice of evidence, interpretation of the evidence, and recommendations for diagnosis and treatment [5],[23]. Some guidelines consider diagnosis of group A β-hemolytic streptococcus essential and consider the prevention of acute rheumatic fever an important reason to prescribe antibiotics. Other guidelines considered acute sore throat a self-limiting disease and do not recommend antibiotics [23].

In most settings in high-income countries such as Norway, the risk of a serious complication arising from using antibiotics for sore throat is of the same order as that of rheumatic fever and suppurative complications of sore throat, all of which are rare [4]. Thus decisions about whether to prescribe antibiotics depend largely on the trade-offs between reducing the duration of symptoms and the downsides of antibiotics, including side effects, the burden of getting and taking antibiotics, and costs [4],[5]. Externalities may also affect decisions, including concerns about spreading infection on the one hand and antibiotic resistance on the other, although the level of evidence for both of these is very low [5].

Thus, in settings where the risk of rheumatic fever and other complications of sore throat are rare, decisions whether to take antibiotics or not are preference sensitive [15],[16]. They depend on the severity of symptoms and the relative importance that individual patients assign to the desirable and undesirable consequences of getting and taking antibiotics. The participants in this study were not a representative sample of the Norwegian population. Nonetheless, the results suggest that many Norwegians would choose not to go to the doctor to get antibiotics for a sore throat (over 75% of participants in this study). However, many patients still do seek medical help for sore throats, and about half of those who do receive a prescription for antibiotics in Norway [17], despite clinical practice guidelines that recommend that patients with sore throat should usually be treated symptomatically without antibiotics [5].

One study showed that while general practitioners and their assistants believed that patients prefer visits to the physician to take tests and receive treatment, rather than have telephone consultations, the patients state that they appreciate evidence-based information about sore throat and the recommendation that testing and consultations were generally not necessary [24]. The results of this study support that finding and recommendations that most patients with sore throat do not need to be seen by a physician and that they should be given good information about the natural history of sore throat and the effects of antibiotics [5]. However, the scenario in the current study addressed the decision of whether to go to the doctor and take penicillin if the doctor recommended it. For patients who go to the doctor for a sore throat, the information given to them should reflect an appropriate diagnosis of whether their sore throat is caused by group A β-hemolytic streptococcus and the increased effectiveness of antibiotics in people with streptococci growing in the throat [4].

The implication of this study for clinicians or others who prepare patient information about antibiotics for sore throat is that this information is more likely to help people to make well-informed decisions if bar graphs of the duration of symptoms are used. Graphical presentations of the proportions of people with sore throat using either bar graphs or face icons are likely to result in decisions that are less consistent with a more fully-informed decision and more people going to the doctor.

### Conclusions

In summary, for people considering whether they should go to the doctor to get antibiotics for sore throat, presenting the benefit of antibiotics in terms of the duration of symptoms appears to help them to make decisions that are most consistent with their own preferences compared to graphical presentations of the proportions of people with sore throat. The extent to which these results can be applied to other decisions is not clear. However, they may be most likely to be relevant when a treatment has a short-term benefit that quickly fades away and relatively important downsides.

## Supporting Information

Figure S1 CONSORT flow diagram.
(0.30 MB TIF)

Text S1 CONSORT checklist.
(0.05 MB DOC)

Text S2 Study protocol.
(0.08 MB DOC)

## Abbreviations

HIPPO: Health Information Project: Presentation Online
OR: odds ratio
RIS: relative importance score(s)
VAS: visual analogue scale(s)

## References

[1] McGettigan P, Sly K, O'Connell D, Hill S, Henry D (1999). The effects of information framing on the practices of physicians.. J Gen Intern Med.

[2] Moxey A, Dip G, O'Connell D, McGettigan P (2003). Describing treatment effects to patients: How they are expressed makes a difference.. J Gen Intern Med.

[3] Epstein RM, Alper BS, Quill TE (2004). Communicating evidence for participatory decision making.. JAMA.

[4] Del Mar CB, Glasziou PP, Spinks AB (2006). Antibiotics for sore throat.. Cochrane Database of Systematic Reviews Issue 4.

[5] Flottorp S, Oxman AD, Cooper JG, Hjortdahl P, Sandberg S (2000). Guidelines for the diagnosis and treatment of sore throat [Norwegian].. Tidsskr Nor Lægeforen.

[6] Wills CE, Holmes-Rovner M (2003). Patient comprehension of information for shared treatment decision making: state of the art and future directions.. Patient Educ Couns.

[7] Guyatt GH, Oxman AD, Kunz R, Flack-Ytter Y, Vist E (2008). Going from evidence to recommendations.. BMJ.

[8] Feldman-Stewart D, Kocovski N, McConnell BA, Brundage MD, Mackillop WJ (2000). Perception of quantitative information for treatment decisions.. Med Decis Making.

[9] Elting LS, Martin CG, Scott BC, Rubenstein EB (1999). Influence of data display formats on physician investigators' decisions to stop clinical trials: prospective trial with repeated measures.. BMJ.

[10] Edwards A (2006). Presenting risk information to people with diabetes. Evaluating effects and preferences for different formats by a web-based randomised controlled trial.. Patient Educ Couns.

[11] Lipkus IM, Hollands JG (1999). The visual communication of risk.. J Natl Cancer Inst Monogr.

[12] Mazur DJ (1990). Interpretation of graphic data by patients in a general medical clinic.. J Gen Int Med.

[13] Janis IL, Mann L (1977). Decision Making: A Psychological Analysis of Conflict, Choice, and Commitment..

[14] Llewellyn-Thomas H  Helping Patients Make Health Care Decisions, Center for the Evaluative Clinical Sciences, Dartmouth Medical School.. http://www.dartmouthatlas.org/atlases/DecisionSupport.pdf.

[15] Wennberg JE, Fisher ES, Skinner JS (2002). Geography and the debate over medicare reform.. http://content.healthaffairs.org/cgi/content/abstract/hlthaff.w2.96v1.

[16] O'Connor AM, Légaré F, Stacey D (2003). Risk communication in practice: the contribution of decision aids.. BMJ.

[17] Flottorp S, Oxman AD, Håvelsrud K, Treweek S, Herrin J (2002). A cluster randomised trial of tailored interventions to improve the management of urinary tract infections and sore throat.. BMJ.

[18] Carling C, Kristoffersen DT, Herrin J, Treweek S, Oxman AD (2008). How should the impact of different presentations of treatment effects on patient choice be evaluated? A pilot randomized trial.. PLoS ONE.

[19] Carling C, Kristoffersen DT, Montori VM, Herrin J, Schünemann HJ (2009). The effect of alternative summary statistics for communicating risk reduction on decisions about taking statins: A randomized trial.. PLoS Med.

[20] Fortin JM, Hirota LK, Bond BE, O'Connor AM, Col NF (2001). Identifying patient preferences for communicating risk estimates: a descriptive pilot study.. BMC Med Inform Decis Mak 1.

[21] Edwards A, Elwyn G, Covey J, Matthews E, Pill R (2001). Presenting risk information: a review of the effects of framing and other manipulations on patient outcomes.. J Health Communication.

[22] Wiseman D, Levin IP (1996). Comparing risky decision making under conditions of real and hypothetical consequences.. Org Behavior Human Dec Proc.

[23] Matthys J, De Meyere M, van Driel ML, De Sutter A (2007). Differences among international pharyngitis guidelines: not just academic.. Ann Fam Med.

[24] Flottorp S, Oxman AD (2003). Identifying barriers and tailoring interventions to improve the management of urinary tract infections and sore throat: a pragmatic study using qualitative methods.. BMC Health Services Research.

[25] Statistics Norway http://www.ssb.no/tabell/03026.

[26] Statistics Norway http://www.ssb.no/tabell/06217.

