# Supplementary figures and images for: The Effect of Alternative Graphical Displays Used to Present the Benefits of Antibiotics for Sore Throat on Decisions about Whether to Seek Treatment: A Randomized Trial
Source: PLoS Med. 2009 Aug 25;6(8):e1000140. doi: 10.1371/journal.pmed.1000140 (PMC2726763; doi:10.1371/journal.pmed.1000140)

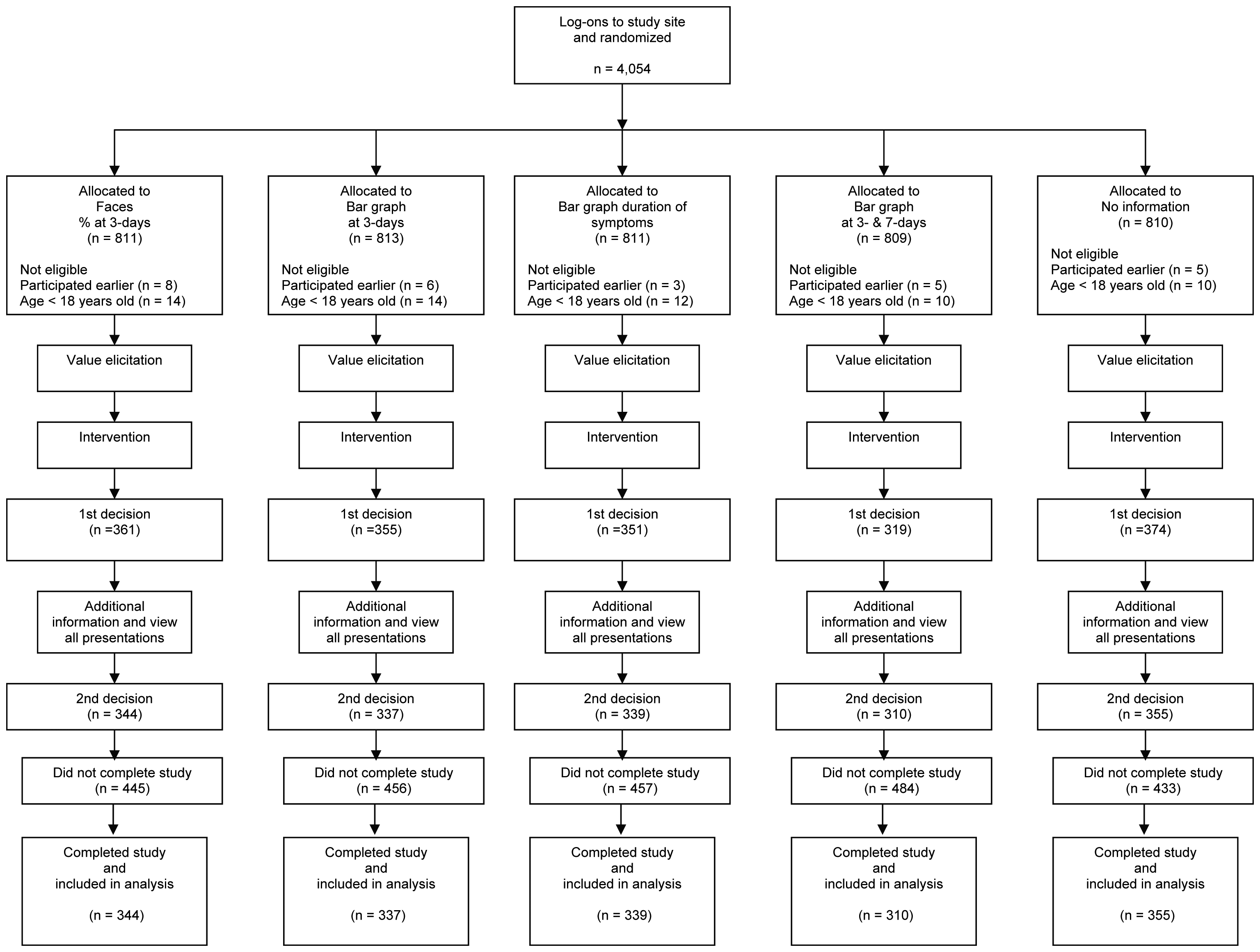

Supplement: Figure S1 — CONSORT flow diagram. (0.30 MB TIF) [file pmed.1000140.s001.tif]
