# Supplementary material for: The Effect of Alternative Graphical Displays Used to Present the Benefits of Antibiotics for Sore Throat on Decisions about Whether to Seek Treatment: A Randomized Trial
Source: PLoS Med. 2009 Aug 25;6(8):e1000140. doi: 10.1371/journal.pmed.1000140 (PMC2726763; doi:10.1371/journal.pmed.1000140)
Supplement: Text S2 — Study protocol. (0.08 MB DOC) [file pmed.1000140.s003.doc]

**The effect of alternative graphical displays used to present the benefits of antibiotics for sore throat on decisions about whether to seek treatment: A randomized trial**

Cheryl Carling, RN, MSc, Research Researcher^1^

Signe Flottorp, MD, PhD, Researcher^1^

Doris Tove Kristoffersen, MSc, Researcher^1^

Andrew David Oxman, MD, Director^1^

Elie Akl, MD, Resident^2^

Phillip J. Deveraux, MD, Research Fellow^3^

Jeph Herrin, PhD, Research Consultant^4^

Tom MacKenzie^5^

Victor Montori, MD, MSc^3,6^

Holger Schünemann, MD, PhD, Assistant Professor^2,3^

1. Informed Choice Research Department

Norwegian Health Services Research Centre, Norway

1. Departments of Medicine and Social and Preventive Medicine

University of Buffalo, USA

1. Department of Clinical Epidemiology and Biostatistics

McMaster University, Canada

1. Flying Butress Associates

Charlottesville, VA, USA

1. Department of Medicine

University of Colorado, USA

1. Knowledge and Encounter Research Unit, Department of Medicine

Mayo Clinic College of Medicine, USA

Address for correspondence:

Informed Choice Research Department

Norwegian Health Services Research Centre

P.O. Box 7004, St. Olavs plass

N-0130 Oslo

Norway

Phone: +47 24 16 32 48

Fax: +47 24 16 30 11

Email: [cheryl.carling@kunnskapssenteret.no](mailto:cheryl.carling@kunnskapssenteret.no)

**BACKGROUND**

Relevant, reliable and accessible information about the effects of interventions is essential for informed choices about healthcare. The manner in which this information is presented affects how it is understood and subsequent decisions.^1-4^ The objective of the Health Information Project: Presentation Online (HIPPO) is to improve communication of information about the effects of healthcare based on randomised trials of alternative ways of presenting this evidence.

The focus of this trial is on the use of graphical presentations of the benefits of treatment for people with sore throat who must decide whether to go to the doctor to get penicillin or another antibiotic.^5,6^ The results of this study will help to inform decisions about how best to communicate the effects of treatment to patients and the general public.

Relatively little previous research has investigated people’s understanding of graphical displays of treatment effects.^7^ Wills et als. review lists 6 studies using graphic displays in a medical decision context : Mazur & Hickham 1993, Mazur & Merz 1993, Mazur & Hickham 1996,Morrow & Hier 1998, Feldman-Stewart et al. 2000, Shapira et al 2001. None of these has investigated the relationship between the graphical display that is used and the extent to which decisions are congruent with expected utilities (EU). Commonly used graphical displays include bar graphs, pie charts, line graphs, and the use of icons, such as happy faces. In a comparison of pie charts, vertical bars, horizontal bars, numbers, systematic ovals, and random ovals for making a choice, vertical bars, horizontal bars, numbers, and systematic ovals were equally well perceived. Pie charts and random ovals led to slower and less accurate performance.^8^

According to Decision Analysis, based on the normative concept of expected utility maximization,^9^ people should choose among options the one which gives the highest expected utility. Expected utility for any choice is calculated by multiplying the probability for each expected outcome by the utility for that outcome, and summing the products for each outcome under that choice option.

EU = Σ p_i_ * u_i_

i

The utility of outcomes, such as different health states, is usually expressed as being between zero and one (e.g. with death having a value of zero and a full healthy life having a value of one).

In this study, we calculate only utilities and disutilities of health states by summation of the utilities of their components. Probability was eliminated from the model because analysis of the pilot data indicated that the improper linear model worked as well as the expected utility model, which is weighted by probabilities.

Expected utility theory has been questioned for a number of reasons, including problems with how utilities are measured and observations that people often do not, in fact, choose to maximize their utilities.^10-14^ Nonetheless, it can still be argued that as the expected utility for a decision, such as to take penicillin for sore throat, increases one would expect that, on average, increasing proportions of people would choose to take the medication, if they are well informed. This argument does not depend on every individual choosing to maximize her utilities. Some people may make decisions based on other factors and it is difficult to accurately measure people’s utilities. Nonetheless, one would expect some degree of correlation between the expected utility of deciding to do something, such as taking pills, and the proportion of people who decide take the pills.

## OBJECTIVES

The objective of this study is to determine which of four ways of presenting information (Figure 1) about the effects of antibiotics on the duration of symptoms for people with sore throat. We have three main comparisons that we will test. Is the congruence between respondents’ utilities and their decision about whether to go to the doctor when they have a sore throat better for a presentation using:

1. icons compared to a bar graph, both displaying the proportion of people who still have sore throat on day three (the point of maximum benefit of antibiotics)?
2. a bar graph displaying the difference in the average duration of symptoms compared to a bar graph displaying of the proportion of people who still have sore throat on day three?
3. a bar graph displaying the proportion of people who still have sore throat on both day three and day seven compared to a bar graph displaying day three only?

## METHODS

Study design

The study is a Web-based randomised trial in which participants are randomised to one of four ways of presenting information about the benefits of antibiotics for sore throat (Figure 1).

We have chosen four presentations based on the information that is reported in a systematic review of antibiotics for sore throat.^5^ Bar graphs are widely used and familiar to most people. The use of icons has become popular more recently, particularly in the context of decision support tools. Arguments for using icons include See Lipkus IM, Hollands JG. The visual communication of risk. J Natl Cancer Inst Monogr 1999;25:149-63.

We initially considered using a line graph (survival curves for the duration of symptoms). This display contains the most information relative to the other alternatives, but following consultations with colleagues, we concluded that this would be difficult for many people to understand. We therefore elected to use a second bar graph presentation that includes similar information to what is presented in a line graph. The fourth display that we chose to include is also a bar graph, but presents the results in terms of the average length of symptoms rather than the proportion of people with symptoms at a specific point in time. Day three and seven were selected as the points in time based on the results of the review. Day three is the point of maximum benefit and by day seven most people no longer have symptoms with or without antibiotics.

Upon logging into the study Website (Appendix), participants will be presented with information about the study and asked to give informed consent to participate. They will view a brief scenario in which they will be asked to imagine that they have a sore throat and they need to decide whether to go to the doctor or not to get penicillin or another antibiotic. Each respondent's utilities for the main benefits and harms will be elicited using a visual analogue scales (VAS) for the importance of each of the following: sore throat, side effects of penicillin, recurrence of sore throat, and the nuisance associated with getting and taking penicillin. They will then be randomly presented with one of the four presentation formats about the effect of antibiotics shortening the duration of symptoms. In order to obtain baseline data, a fifth group will not be given any information. Participants will then be asked whether they would decide to go to the doctor or not. Thereafter, respondents will be shown all four presentations and provided with more detailed patient information. They will be asked a few questions about themselves, their preferred presentation format among the four, and they will be asked to reconsider their decision.

Inclusion criteria

Only complete responses from persons who where at least 18 years old and literate in the language that they selected to participate in the study will be included in the analysis.

Participants will be recruited in Norway through a nationally broadcast weekly health television program, PULS. In introducing the study, viewers will be shown the results of a study documenting wide variation in the use of antibiotics for sore throat in Norway, asked what they do when they have a sore throat, and invited to go to the programs web pages to obtain more information and to find out whether how this information is presented affects the choices that people make. From the PULS website viewers will be referred to the HIPPO website using a link.

Allocation

Allocation to one of the four presentations or no presentation will be determined by block-randomization. We created a sequence of 400 presentation assignments by generating 100 blocks of the four presentations using [http://www.randomization.com](http://www.randomization.com/) (Randomization plan created on 17 April 2002, 16:03:41. To reproduce this plan, use the seed 11049). This sequence will be looped and repeated.

Data collection

Participants enter responses directly by responding to the questions on the HIPPO website. The data generated will be stored anonymously in a database. Only completed records will be used in the main analyses.

Analysis

We have the following variables:

U_S_ = utility of having the **symptoms** of a sore throat; i.e. 1 – how difficult it is having the symptoms of a sore throat on a scale from 0 to 1; 1 equals the most difficult

U_A_ = utility of the **adverse effects** of penicillin; i.e. 1 – how difficult it would be to have one of the common adverse effects of penicillin on a scale from 0 to 1; 1 equals the most difficult

U_R_ = utility of a **recurrence** of sore throat; i.e. 1 – how difficult it would be to have another sore throat in the next few months on a scale from 0 to 1; 1 equals the most difficult

U_N_ = utility of the **nuisance** of getting and taking penicillin; i.e 1 – how difficult it would be to get and take antibiotics on a scale from 0 to 1; 1 equals the most difficult

The disutility is given by:

S = (1-U_A_) + (1-U_R_) + (1-U_n_) - (1-U_S_) = VAS_A_ + VAS_R_ + VAS_N_ – VAS_S_

The main objective of the study will be to identify which of the four presentations results in decisions that are most consistent with what individuals would be expected to decide based on their utilities. That is, we would expect individuals having high disutility value (S) to decide not to go to the doctor and persons with a low disutility value to go to the doctor. The data will be analysed using a binary logistic regression model to identify which of the summary statistics result in the above. If G is used to represent the presentation group to which a participant is randomised and D represents the decision to go to the doctor or not, then the relationship between D, G and S, can be modelled as follows:

(Eq 1)          logit(D) = β_0_ + β_1g_G_g_ + β_2_S + β_3g_G_g_*S; g=1,..,4

If there is a difference between the presentations, G will be a significant variable in the model. When evaluating the decision to go to the doctor, the probability should have a fairly high value for people who rank their disutility of having sore throat much higher than their sum of the other disutilities, whereas having more concern about adverse effects, recurrence and the nuisance of getting and taking penicillin should be reflected in a low probability (low predicted value). Thus, one might think that the presentation group with the steepest slope should be identified as the group providing the best congruence between respondents’ utilities and their treatment choice. This however, will not be the most reliable result for a group if it has a very high proportion of individuals deciding to go to the doctor independent of the utility scores.

The slopes estimated from Eq 1, will be compared in order to test the hypothesis of no difference in slopes between the groups. The main pairwise comparisons in which we are interested are:

1. icons compared to a bar graph, both displaying the proportion of people who still have sore throat on day three (the point of maximum benefit of antibiotics)
2. a bar graph displaying of the difference in the average duration of symptoms compared to a bar graph displaying of the proportion of people who still have sore throat on day three
3. a bar graph displaying the proportion of people who still have sore throat on both day three and day seven compared to a bar graph displaying day three only

No adjustment will be made for multiple testing for these three comparisons. The primary analysis will compare the slopes in an analysis of variance and the pairwise comparisons. We will also consider the intercepts in interpreting the results; i.e. whether any of the presentations result in a very high proportion of people deciding to go to the doctor, or deciding not to go, independent of their disutility value. A Bonferroni correction will be used for any other pairwise comparisons that are made.

Sample size

The results from logistic regression of data collected for a previous study have been used to obtain estimates to use for sample size calculations. The number of subjects to be included in each group is based on power calculations for obtaining a statistically significant difference in slopes between the groups. Based on the differences in slopes observed in an earlier study, a significance level of 0.05 and 80% power we estimate that we will need 600 participants per group.

Additional analysis

Additional questions that this study will address are:

- - Which presentation results in decisions that are most consistent with “fully informed” choices, after participants have seen all four presentations and been provided more detailed information?
  - Which presentation do participants prefer?

## Ethics

The protocol will be submitted for approval to the Norwegian Data Protection Agency and the Regional Ethics Committee. The interventions in this study, alternative ways of presenting information on the effects of treatment, and asking participants to make a hypothetical decision, are none-invasive and harmless. The same information on the benefits and harms of penicillin is widely available and is presented in many different ways, including those used in the study.

Participants are informed on the consent screen that they can leave the study at any time, and are given the option of choosing to have any data that they might have entered deleted.

Confidentiality of data entered by participants is ensured by not collecting any information that would make it possible to identify participants. If participants fill out forms to indicate an interest in participating in future studies, their email addresses will not be stored in the same database as their responses nor will these databases be linked in any way.

## REFERENCES

1. McGettigan P, Sly K, O’Connell D, Hill S, Henry D. The effects of information framing on the practices of physicians. J Gen Intern Med. 1999; 14:633-42.
2. Herrin J, Schünemann H, Oxman AD, Vist G, Olsen K. Presentation of empirical evidence about health (Cochrane Review). In: The Cochrane Library. Oxford: Update Software. Under revision.
3. Moxey A, Dip G, O’Connell D, McGettigan P. Describing treatment effects to patients: How they are expressed makes a difference. J Gen Intern Med 2003;18:948-959.
4. Epstein RM, Alper BS, Quill TE. Communicating evidence for participatory decision making. JAMA 2004;291:2359-2366.
5. Del Mar CB, Glasziou PP, Spinks AS. Antibiotics for sore throat (Cochrane Review). In: The Cochrane Library, Issue 2, 2004.
6. Flottorp S, Oxman AD, Cooper JG, Hjortdahl P, Sandberg S, Vorland LH. Retningslinjer for diagnostikk of behanling av sår hals. Tidsskr Nor Lægeforen 2000;120:1754-60.
7. Wills CE, Holmes-Rovner M. Patient comprehension of information for shared treatment decision making: state of the art and future directions. Patient education and Counseling 2003;50:285-290.
8. Feldman-Stewart D, Kocovski N, McConnell BA, Brundage MD, Mackillop WJ. Perception of quantitative information for treatment decisions. Med Decis Making 2000;20:228-238.
9. Von Neumann J,Morgenstern O. Theory of Games and Economic Behavior. New York: Wiley, 1944.
10. Schoemaker PJH. The expected utility model: its variants, purposes, evidence and limitations. J Economic Literature 1982;20:529-35.
11. Llewellyn-Thomas H, Sutherland HJ, Tibshirani R, et al. The measurement of patients’ values in medicine. Med Decision Making 1982; 2:449-62.
12. Hellinger FJ. Expected utility theory and risky choices with health outcomes. Med Care 1989; 27:273-9.
13. Frisch D, Clemen RT. Beyond expected utility: rethinking behavioral decision research. Psychol Bull 1994; 116:46-54.
14. Schwartz S, Griffin T. Medical Thinking: the Psychology of Medical Judgment and Decision Making. New York: Springer-Verlag 1986; 13.
15. Skolbekken JA. Communicating the risk reduction achieved by cholesterol reducing drugs. BMJ 1998; 316:1956-8.
16. Hollnagel H. On the language of risk in the medical consultation [Danish]. Practicus 1996; 116:237-9.
17. Cohen J. Statistical Power Analysis for the Behavioral Sciences. Second Edition. Lawrence Erlabaum Associates.
18. Dawes RM. The robust beauty of improper linear models in decision making. American Psychologist 1979, Vol. 34, No.7, 571-582.
